# Development of the C4 inventory: a measure of common characteristics that complicate care in outpatient psychiatry

**DOI:** 10.15256/joc.2016.6.66

**Published:** 2016-05-25

**Authors:** Robert G. Maunder, Lesley Wiesenfeld, Sian Rawkins, Jamie Park

**Affiliations:** ^1^Mount Sinai Hospital, Toronto, Ontario, Canada; ^2^Department of Psychiatry, University of Toronto, Toronto, Ontario, Canada; ^3^Li Ka Shing Knowledge Institute, St. Michael’s Hospital, Toronto, Ontario, Canada

**Keywords:** Comorbidity, mental disorders, substance-related disorders, social determinants of health, child abuse, checklist

## Abstract

**Background:**

Psychiatric syndromes are complicated by comorbidity and other factors that burden patients, making guideline-informed psychiatric care challenging, and negatively affecting outcome. A comprehensive intake tool could improve the quality of care. Existing tools to quantify these characteristics do not identify specific complications and may not be sensitive to phenomena that are common in psychiatric outpatients.

**Objective:**

To develop a practical inventory to capture observations related to complex care in psychiatric outpatients and quantify the overall burden of complicating factors.

**Design:**

We developed a checklist inventory through literature review and clinical experience. The inventory was tested and compared with related measures in a cross-sectional study of 410 consenting outpatients at the time of initial assessment.

**Results:**

The summed score of inventory checklist items was significantly correlated with patient-assessed measures of distress (K10, *r*=0.36) and function (WHODAS 2.0, *r*=0.31), and physician-assessed measures of function (GAF, *r*=−0.42), number of psychiatric diagnoses [*F*(df3)=33.6], and most complex diagnosis [*F*(df3)=37.4]. In 53 patients whose assessment was observed by two clinicians, inter-rater reliability was acceptable for both total inventory score (intraclass correlation, single measures = 0.74) and agreement on specific items (mean agreement score = 90%).

**Conclusions:**

The Psychiatric C4 Inventory is a reliable instrument for psychiatrists that captures information that may be useful for quality improvement and resource planning. It demonstrates convergent validity with measures of patient distress, function, and complexity. Further tests of validity and replication in other settings are warranted.

## Introduction

The most prevalent psychiatric syndromes treated in ambulatory settings are mood disorders, anxiety disorders, and substance use disorders [1]. These syndromes are commonly complicated by concurrent medical [2] and psychiatric disorders [1]. It is also common for patients with these syndromes to be ­treatment-resistant [3–5] or to experience symptoms and safety risks, such as suicidality or psychosis, which require additional management strategies, including hospitalization. These phenomena increase the burden for patients, tend to make psychiatric care more complicated, and negatively affect prognosis. In clinical trials of psychiatric treatments, complicating aspects of psychiatric or psychosocial comorbidity are often exclusion criteria. Thus, evidence-based interventions and treatment guidelines tend to focus on discrete diagnoses, and may not take the breadth of common inter-related clinical challenges into account. As a result, guidelines rarely provide direction regarding strategies (such as treatment sequencing) to deal with comorbidities and complex psychosocial circumstances. This gap between efficacy and effectiveness could act as a barrier to evidence-based treatment planning and optimal resource allocation.

### Identifying relevant characteristics at the time of assessment

In our outpatient center, our goal was to identify and quantify the characteristics that commonly complicate the treatment of mental illness in order to develop and adapt treatment resources to meet our patients’ needs. Specifically, we wanted to capture information that is assessed during a routine psychiatric intake assessment in a form that allowed us to understand the characteristics and needs of large sets of patients (e.g. all outpatients, or all patients at a certain clinic). The characteristics of interest are those that increase difficulty of diagnosis, increase the amount of resources that are required to obtain a satisfactory patient outcome (e.g. intensity or duration of intervention, number of interventions), or decrease the quality of outcome that can be obtained with standard treatment.

Identifying the characteristics of patients and their circumstances that complicate psychiatric care may be valuable when organizing mental health services. For example, understanding the real-life issues that need to be addressed in a patient’s treatment informs decisions about their access to different interventions or different levels of stepped care, particularly when collaborative care is shared between primary care providers and psychiatrists [6–8]. A valid measure to systematically collect these characteristics could enhance the decision-making around the optimal distribution of mental health resources.

It is a challenge to measure characteristics of complex care because they are very heterogeneous. While it is common for substance abuse and comorbid psychiatric and medical diagnoses to co-occur [9, 10], there are also other psychosocial contributors to complicated psychiatric presentations, including poverty [11, 12], exposure to psychological trauma [13], and maladaptive personality traits [14]. For purposes of treatment planning and resource development, an ideal measure of these phenomena would estimate the overall weight of complicating factors *in addition to* identifying the particular characteristics that complicate care.

### Available instruments

A second challenge is that existing measures are not designed for treatment resource planning in ambulatory settings. There are several instruments available to measure overall levels of function or impairment, including the Global Assessment of Functioning (GAF) scale [15], the Health of the Nation Outcomes Scale (HoNOS) [16], and the World Health Organization Disability Assessment Schedule (WHODAS) [17]. There are also instruments that assess the overall weight of some of these characteristics to facilitate appropriate triage or referral, such as the Threshold Assessment Grid (TAG) [18] and the INTERMED complexity assessment grid [19]. These instruments are valuable for evaluating the level of need for intense or hospital-based resources. However, because they do not identify a wide range of specific patient characteristics, they are less useful for identifying the need for developing new resources. For example, if patients at a mood disorders clinic had an unmet need for addiction services or trauma-focused care, it would not be identified by these measures. Furthermore, some of these instruments (e.g. TAG) lay emphasis on the most severe sources of complicated care (e.g. harm to self and others, threats to survival) and may be insensitive to less severe but more common problems.

There are also many instruments that measure a single component of psychiatric complexity (such as suicidality or exposure to psychological trauma). These are useful to identify and evaluate the specific constructs in individuals, but it would be unwieldy to use standard instruments to simultaneously assess many specific characteristics in this way in routine clinical practice. Similarly, the existing tools that attempt to comprehensively capture characteristics of this kind in inpatients, such as the Resident Assessment Instrument-Mental Health [20], are very time-consuming and would be impractical in ambulatory settings.

Our review of existing instruments indicates that they are not promising for capturing the desired information. However, standard clinical assessment interviews routinely identify a wide range of relevant characteristics. Thus, the challenge may not be to develop new tools that identify these characteristics, but rather to develop tools that can efficiently harvest the information that is already acquired in clinical interviews. Once harvested, an individual’s characteristics can contribute to improvements in systems of care through quality improvement, program development, and resource allocation. Thus, we chose to develop an instrument that efficiently captures information acquired during psychiatric assessment interviews.

### Objective

Our objective was to create an efficient tool that could be used in a psychiatric ambulatory setting at the point of intake assessment, that aligned with a clinically oriented assessment (i.e. not a research assessment), and that required no special training. The purposes of this paper are to describe the development of an instrument that documents and quantifies *common characteristics that complicate care* (C4) in psychiatric outpatients, the Psychiatric C4 Inventory, to report the initial tests of its reliability and validity, and to describe the prevalence of these characteristics among outpatients assessed in the psychiatric department of a general hospital.

## Methods

### Instrument development

We developed an inventory that does not alter the structure of the psychiatric assessment interview as it is typically performed by a psychiatrist or psychiatric resident. The inventory was designed to be completed by a psychiatrist and/or supervised psychiatric resident trainee after an assessment interview with a new patient in order to quickly record the presence or absence of a large number of potential correlates of complicated psychiatric care. The intent was that the tool would yield both item-level information (allowing measurement of the prevalence of particular characteristics) and a summary weight of the multiplicity of characteristics, calculated as the sum of items endorsed.

In developing items for the first draft of the inventory, we were guided by the literature regarding the characteristics of patients and their circumstances that are associated with severe and persistent mental illness and with treatment-resistance [11–14, 21–23]. Additional items were added through the authors’ clinical experience of characteristics that are both common and associated with difficulty in making a diagnosis, higher than typical intensity or duration of intervention, greater number of interventions, or poorer than typical outcomes of treatment. Other psychiatrists in our department were asked to review a list of proposed items to suggest missing characteristics. The practical need for brevity was operationalized as the constraint that the inventory must not exceed a single page. To accomplish this, we collapsed related characteristics into a single item (e.g. “chronic pain, current opiate analgesics or methadone”) rather than creating multiple items. We avoided creating multiple items that were sufficiently closely related that a single patient circumstance would be counted twice because we intended to sum the number of items endorsed as an overall “weight” of complexity.

Some items referred to criteria that were clearly categorical (e.g. “admitted to hospital for mental health reason in past 2 years”), while others required a threshold that involved judgment (e.g. “potentially harmful impulsivity”). In order to facilitate an inventory in a simple checklist format, the threshold for the latter items was set as a characteristic “which complicates assessment or treatment” in the opinion of the assessing clinician.

A 38-item version of the instrument was drafted and pilot tested by 13 psychiatrists who completed the inventory after assessment interviews with a convenience sample of 74 outpatients referred for psychiatric consultation. The psychiatrists were asked to identify items that were ambiguous or difficult to apply, and to suggest modifications and new items that would capture common characteristics of complicated care that were missing from the inventory. The participating psychiatrists then met as a group to review and discuss the aggregate results to form a consensus about suggested changes.

Based on the pilot experience, the inventory was modified in the following ways: items that were rarely endorsed were removed, items that were similar were collapsed into composite items, wording was changed to decrease ambiguity, and an “other” category was added to allow clinicians to identify characteristics of complicated care that are not otherwise captured in the checklist. The determination that items were similar enough to collapse was made based on the consensus of participating psychiatrists evaluating the items’ face meaning. The process of revision resulted in a checklist of 33 specified items, plus 2 “other” (write-in) items, which yielded a summary score between 0 and 35. Items that were judged to be present were checked on the inventory. Items that were absent or unknown were left unchecked. Thus, unchecked items were not considered to be missing data. When no items were present, “none of the above” was to be checked in order to distinguish a record of a patient who had no identified characteristics from an inventory that had not been completed.

### Instrument evaluation

#### Study population

The inventory was completed for consecutive patients assessed in the general ambulatory assessment clinic and outpatient consultation–liaison assessments in the department of psychiatry of Mount Sinai Hospital, Toronto, Canada. All outpatients assessed by participating psychiatrists were eligible and all consenting patients were included. Data were collected between January and November 2014, with the sampling period intended to capture a representative range of patient presentations, rather than targeting a specific sample size. In total, 749 patients were assessed during the study period. Standard measures were used to collect data from 611 (81.6%) of these patients. Of those for whom standardized data were available, 410 (67%) consented to their data being used for research purposes; these patients formed the cohort for this study. This study was approved by the Mount Sinai Hospital Research Ethics Board.

The 35-item Psychiatric C4 Inventory was included with standard measures used routinely for outpatients referred to the department at the time of their first assessment interview. The other instruments were measures completed by patients (demographic data, K10 [24] to measure psychological distress, and WHODAS 2.0 [17] to measure disability) and by clinicians (psychiatric and medical diagnoses as determined by psychiatrists, GAF). Diagnoses were recorded as the presence or absence of each of the 12 diagnostic groups, derived from chapters of DSM-5 [Depressive Disorders, Bipolar and Related Disorders, Anxiety Disorders, Schizophrenia Spectrum and Other Psychotic Disorders, Substance-related and Addictive Disorders, Trauma and Stressor-related Disorders (includes Posttraumatic Stress Disorder, does not include Adjustment Disorder), Somatic and Related Disorders, Dementia/Major Neurocognitive Disorder, Delirium, Personality Disorders, Adjustment Disorder, Other Psychiatric Disorder] or no psychiatric disorder.

### Analysis

#### Reliability

Tests of internal consistency (e.g. Cronbach’s alpha) are not applicable to an inventory of heterogeneous items. Inter-rater reliability was assessed in interviews for which two clinicians (staff psychiatrist observer and psychiatric resident interviewer) were present. The resident and the staff psychiatrist independently completed the inventory, blinded to each other’s evaluation. Inter-rater reliability was tested in two ways. First, the sum of inventory items endorsed was compared between raters using an intraclass coefficient. Second, because identical scores can be generated by different combinations of items, an agreement score was calculated for each pair of ratings (total number of items for which the two raters agreed, expressed as a percentage of all items).

#### Validity

Inventory summary scores were compared with other assessments that are related to the construct of complicated psychiatric care. It was hypothesized that higher inventory summary scores would be associated with (i) lower GAF ratings, (ii) higher number of concurrent psychiatric diagnoses (omitting the checklist item for “3 or more psychiatric diagnoses” from this analysis), (iii) greater WHODAS 2.0 score, (iv) greater K10 score, and (v) “most complicated” psychiatric diagnosis.

In order to designate a “most complicated” psychiatric diagnosis for patients with more than one diagnosis, we generated a list of the diagnostic groups listed above, to be ranked by their likelihood of involving complicated psychiatric care (in general, not with respect to specific patients). Fifteen psychiatrists were asked to rank these diagnostic groups according to “difficulty or complexity that includes the challenge of confidently identifying and formulating psychiatric problems, the intensity of treatment required (high cost resources, number of clinicians, frequency of contact, duration of treatment), the likelihood of tension or strained or ruptured alliance, and the likelihood of multiple treatment trials with unsatisfactory outcomes.” Based on the results, diagnostic categories were ranked from most to least likely to involve complicated care, and the category with the highest ranking for a particular patient was assigned as the patient’s “most complicated” diagnosis (i.e. assessing psychiatrists were not asked to rank diagnoses for individual patients). For the sake of comparison with inventory summary scores, “most complicated” diagnoses were sorted into three categories; highest degree of complication (post-traumatic stress disorder, personality disorder, schizophrenia, bipolar disorder, addiction), moderate degree of complication (depressive disorders, anxiety disorder), and lowest degree of complication (adjustment disorder, no diagnosis). For this comparison “other psychiatric diagnosis” and categories for which the expert rankings were inconsistent (dementia, delirium, somatic disorders) were excluded.

Inventory summary scores were compared with other assessment scores using Spearman’s correlations, paired samples *t*-tests, and analysis of variance, as appropriate. Statistics were computed using IBM SPSS Statistics, version 22. Statistical significance was set at *p* <0.05 (two-tailed).

## Results

The characteristics of 410 patients for whom the Psychiatric C4 Inventory was completed are presented in Table 1. Median age was 39 years (range 18–71 years, interquartile range 28–50 years). The summary score of endorsed items (possible range 0–35) ranged from 0 to 17. The median score was 5 and the interquartile range was 3–7.

**Table 1:** Patient characteristics.

	Number	Percent
Gender		
Male	183	44.6
Female	226	55.1
Missing	1	0.2
Assessment clinic		
General psychiatry ambulatory assessment	292	71.2
HIV Psychiatry	74	18.0
Ambulatory consultation–liaison	44	10.7
Diagnoses		
Depressive disorders	239	58.3
Anxiety and obsessive–compulsive disorders	155	37.8
Substance use disorders	61	14.9
Post-traumatic stress disorder	41	10.0
Personality disorders	35	8.5
Bipolar and related	28	6.8
Somatic disorders	18	4.4
Adjustment disorder	16	3.9
Schizophrenia and related	14	3.4
Other diagnosis	39	9.5
No diagnosis	8	2.0
No diagnostic information	19	4.6
Number of diagnostic categories present		
One	195	47.6
Two	138	33.7
Three	48	11.7
Four	10	2.4
No diagnostic information	19	4.6

HIV, human immunodeficiency virus.

The frequency with which individual inventory items were endorsed is presented in Table 2. The “other” category was endorsed in four patients assessed (1%). Items written in the “other category” were: “caregiver burden for family members with severe mental illness,” “learning disability,” “medical illness in childhood with multiple hospitalizations,” and “minimizing, suppressing symptoms and avoiding treatment.”

**Table 2:** Psychiatric C4 Inventory items sorted by frequency of endorsement.

	Number	Percent
More than two episodes of episodic illness (e.g. major depression) OR >2 years of continuous illness	235	57.3
Any childhood abuse or neglect	148	36.1
Internalizing traits (shame, worthlessness, excessive guilt)	146	35.6
Physical health condition	146	35.6
Lack of affiliation trait (excessive distancing, isolation, interpersonal avoidance)	125	30.5
Interpersonal isolation, actual/perceived lack of support, interpersonal conflict (e.g. divorce)	119	29.0
Self-critical perfectionism trait	119	29.0
Clinically significant adult trauma or adversity	93	22.7
Significant childhood loss (e.g. death of a parent, sibling) or permanent separation of parents	84	20.5
Problematic pattern of alcohol or drug use (prescription or nonprescription)	83	20.2
Three or more psychiatric diagnoses	75	18.3
Admitted to hospital for mental illness in past 2 years	73	17.8
Catastrophizing trait	70	17.1
Racism, discrimination or stigma	62	16.1
Chronic pain, current opiate analgesics or methadone	62	15.1
Treatment resistance (e.g. >two trials of different treatments)	57	13.9
On disability support or unemployed due to mental illness in past 2 years	50	12.2
Clinically significant losses as an adult (e.g. multiple deaths, death of a child)	49	12.0
Inadequate housing, financial situation, poverty	51	12.4
Lack of agency (passive, lack of problem solving or initiative)	48	11.7
Potentially harmful impulsivity	47	11.5
Multiple assessments by different mental health clinicians in past 2 years	44	10.7
Lack of independence trait (excessive dependence, help-seeking, clinging)	33	8.0
Self-harm or suicide attempt in past 2 years	28	6.8
Psychosis or mania in past 2 years	21	5.1
Strong negative expectation about effectiveness of treatment	21	5.1
Barriers to treatment engagement or motivation (e.g. coerced, accommodating someone else’s need, significant disagreement about the nature of problems or the goals of treatment)	19	4.6
Language or cultural barriers	16	3.9
Geographic barriers/lack of local resources	10	2.4
Intellectual impairment or cognitive deficit	10	2.4
Current active legal involvement	8	2.0
Violence/harm to others in past 2 years	3	0.7
Sociopathy trait	1	0.2

### Inter-rater reliability

Fifty-three assessment interviews were observed by two clinicians (resident and staff psychiatrist). The intraclass correlation for comparison of summary score results between the two clinicians (single measures statistic) was 0.74 (95% confidence interval 0.59–0.84, *p*<0.001). The difference between staff psychiatrists’ inventory summary scores (mean 5.0) and residents’ inventory summary scores (mean 5.4) was not significant (paired *t*-test, *p*=0.18).

At the level of individual checklist items, mean item agreement between raters (possible range 0–35) was 31.5 (90%, mode 32, median 32, range 24–35). Item level inter-rater reliability of inferential items (possible range 0–24) was 21.4 (89%) and for non-inferential items (possible range 0–11) was 10.0 (91%).

### Comparison with related measures

**Figure 1:**
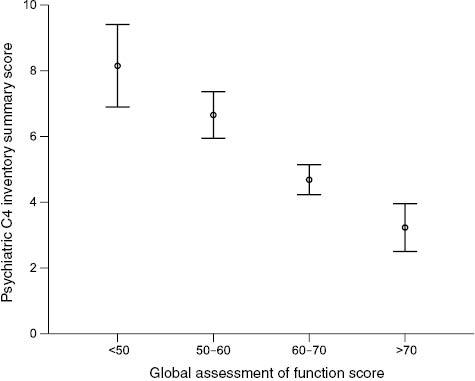
Psychiatric C4 inventory summary score compared with the global assessment of functioning score. Values are means with 95% confidence intervals.

**Figure 2:**
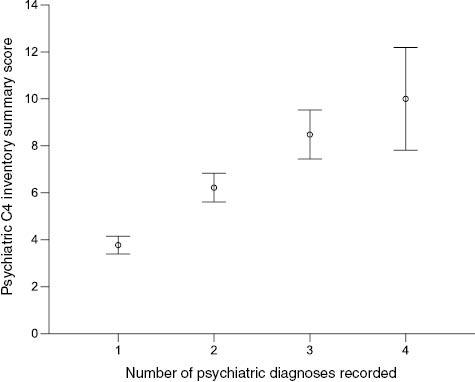
Psychiatric C4 inventory summary score compared with the number of psychiatric diagnoses recorded. Values are means with 95% confidence intervals.

**Figure 3:**
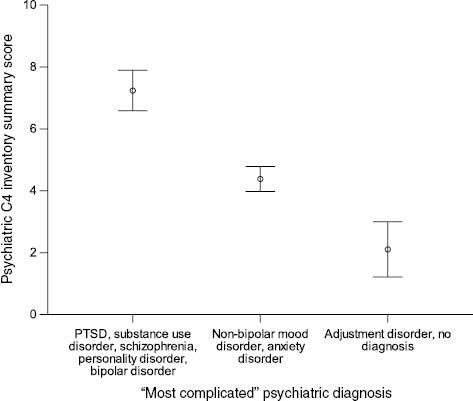
Psychiatric C4 inventory summary score compared with the “most complicated” diagnosis recorded after assessment. Values are means with 95% confidence intervals. PTSD, post-traumatic stress disorder.

The correlation between inventory summary scores and GAF scores was moderately strong and significant (Spearman’s rho = −0.44, *p*<0.001, missing data: 19). The relationship is illustrated in Figure 1. The relationship between number of psychiatric diagnoses recorded and inventory summary scores was also significant [*F*(df3)=33.6, *p*<0.001, missing data: 21] and is illustrated in Figure 2. Inventory summary scores were positively correlated with K10 scores (*r*=0.36, *p*<0.001, missing data: 1) and WHODAS 2.0 scores (*r*=0.31, *p*<0.001, missing data: 1). Inventory summary scores also differed significantly by “most complicated” psychiatric diagnosis [*F*(df3)=37.4, *p*<0.001, missing data: 56], as is illustrated in Figure 3.

## Discussion

We describe the development of an instrument designed to capture characteristics that are associated with complicated psychiatric care, as well as to compute a summary weight of these characteristics for patients assessed by psychiatrists or trainees in psychiatry. The Psychiatric C4 Inventory has several characteristics of a practical tool. It does not require a structured interview or any additional assessment beyond the clinical norm. The results presented in this study are based on using the instrument without any training being given to the raters on its use. It is also brief; informal feedback from clinicians suggests that it is typically completed in about 1 minute following an assessment interview.

Inter-rater reliability is adequate for a tool that is not intended for clinical decision-making in individuals (intraclass correlation, single measures = 0.74). Since the two raters differed in clinical experience in this study, it is likely that reliability would be higher among experienced raters. Although overall reliability was acceptable, there was a weak trend towards lower reliability on items that require an inference about clinical importance. This suggests that inter-rater reliability could be further improved, if necessary, by training to gain consensus around the clinical threshold for determining whether or not a characteristic is likely to “complicate assessment or treatment.”

Tests of convergent validity with a physician-assessed measure (GAF) and with patient-assessed measures (K10, WHODAS 2.0) were all significant and moderately strong. The overall inventory summary score was also significantly related to clinically assessed markers of potentially complex care requirements, such as the number of psychiatric diagnoses and the presence of diagnoses that are generally considered by clinicians to require more complex care. Thus, the validity of Psychiatric C4 Inventory is supported by its relationship with measures that use various methods of assessment.

### Potential benefits

The potential benefit of capturing individual components of complicated care is the ability to use that information to assess the appropriateness of existing resources and plan the development and organization of new treatment resources. For example, in the cohort of patients assessed in this setting, childhood abuse and neglect and interpersonal isolation were among the most common complicating characteristics (see Table 2). Identifying these common sources of complexity may indicate the value of developing new treatment resources or new referral relationships for characteristics that are not currently well-addressed. For example, it may improve patient care to consider how a therapeutic focus on the consequences of childhood trauma could be incorporated into various types of treatment across diagnoses. This might require the development of new treatment programs and/or the development of trauma-focused treatment skills by professionals within existing programs. As another example, a specific focus on reducing interpersonal isolation might improve treatment outcomes and improve patients’ quality of life. This could be accomplished by increasing referrals to community resources that strengthen interpersonal support or reduce barriers to support, or by reinforcing and emphasizing the teaching of interpersonal skills within existing programs. While there are many potential solutions, none can be tried and tested until the need is identified, which is the role of the Psychiatric C4 Inventory.

The Psychiatric C4 Inventory is not primarily designed as an aid to individual treatment planning, but it may prove to be useful for this purpose. Although the information that it summarizes is known to the assessing clinician, by drawing attention to key foci, the inventory may serve as a reminder of important clinical issues at the end of an assessment, which may be valuable in considering potential interventions.

### Limitations and special considerations

Correlates of complicated care are likely to vary between different cohorts of patients and in different treatment settings. A forensic clinic would be likely to identify more sociopathy, a clinic for severe and persistent mental illness would identify more psychosis, and so forth. As such, the selection of items in the inventory may be biased towards the characteristics of the patients in the setting where it was developed. It is likely that some settings will prefer to add items to the Psychiatric C4 Inventory in order to capture sources of complicated care that are common in their patients. For example, discussion with outpatient geriatric psychiatrists led to the suggestion to add items that are relevant for their patients: “harm or abuse from another person,” “incapacity to make medical decisions or manage finances,” “impaired ability to perform basic activities of daily living,” and “family/carers unable to meet patients’ needs.” One of the intended purposes for the “other” category of write-in items is to provide a means by which to capture important characteristics that are not included in the inventory. If a particular characteristic is frequently endorsed in the “other” category, consideration should be given to adding it to the inventory. Of course, adding items of local relevance prevents the use of inventory summary scores to make comparisons between different settings. The very low rate of endorsement of the “other” category in this study suggests either that the inventory’s items were deemed sufficient for the setting in which it was tested, or that training is required to encourage clinicians to use the “other” category when needed.

The overall inventory summary score is affected by the relative weighting of items, which in this analysis is assumed to be equivalent (a simple sum of 1 point for each endorsed item). Individually weighting items would require further research comparing the individual contribution of particular items to an independently determined outcome (for example, markers of a complicated course of treatment collected prospectively). It is possible that calculated item-weights could yield an overall score with greater validity, but this would also make the use of the inventory more cumbersome. Also of relevance to the issue of the weight of certain characteristics, some items on the inventory potentially overlap with each other. This concern may apply to “lack of affiliation” (which is intended to capture a trait: clinically significant attachment avoidance) and interpersonal isolation (intended to capture an actual lack of integration or support). In this example in the current study, co-endorsement was not the rule but was common: 53% of patients for whom lack of affiliation was endorsed were also judged to be interpersonally isolated; 56% of patients who were judged to be interpersonally isolated were also described as having the lack of affiliation trait. Concerns about possible over-weighting and under-weighting markers of complexity (through including multiple similar items, or collapsed items) should be addressed in future studies of the ability of the Psychiatric C4 Inventory to predict relevant patient outcomes. The inventory may also provide data by which to assess the importance of interactions between various items, which are not accounted for by simply summing checked items. The method for developing the Psychiatric C4 Inventory is limited by collapsing items based on consensus, rather than analytically, using confirmatory factor analysis.

### Next steps

Further tests of validation are necessary. Prospective tests of predictive validity in patients entering psychiatric treatment are required to test if the Psychiatric C4 Inventory captures markers of complexity that actually compromise treatment outcomes. Similarly, since the inventory is intended to identify patients who need more resource-intense interventions, prospective tests of the relationship between inventory summary scores at assessment and subsequent referral patterns would be useful. At the item level, the validity of individual items could be tested by comparing the endorsement of items with validated measures of the relevant constructs. Each of these validation strategies is currently underway.

Further research could focus on the potential that particular clusters of complexity items are of value in treatment planning, in predicting prognosis or for other clinical purposes. Such a use of the Psychiatric C4 Inventory would align with studies that show, for example, that pre-treatment data, including marital status, employment status, life events, comorbid personality disorder, and prior medication trials, can predict preferential response to different treatments for major depression [25].

There are limits on the utility of the Psychiatric C4 Inventory in practice. The most substantial of these is that its breadth of data capture and reliability depend on the assumption that an adequate psychiatric assessment interview has been performed. In this study, assessment interviews were performed by psychiatrists and supervised psychiatric residents, which is the norm in our setting. Thus, the reliability of the inventory when used by non-physician mental health professionals requires further study, and the inventory may not be applicable in settings where more limited or focused assessment is standard (for example, in emergency assessment). The need for complete psychiatric assessment also limits the possibilities for using the inventory to determine how characteristics change over time.

In summary, pending replication of these findings in other settings and of further tests of validity, the Psychiatric C4 Inventory has the characteristics of a practical tool that allows psychiatrists to quickly harvest information about the types of characteristics that contribute to complex care and the overall burden of complexity in psychiatric outpatients.
